# Genetically Engineered Yeast Expressing a Lytic Peptide from Bee Venom (Melittin) Kills Symbiotic Protozoa in the Gut of Formosan Subterranean Termites

**DOI:** 10.1371/journal.pone.0151675

**Published:** 2016-03-17

**Authors:** Claudia Husseneder, Jennifer R. Donaldson, Lane D. Foil

**Affiliations:** Department of Entomology, Louisiana State University Agricultural Center, Baton Rouge, Louisiana, United States of America; Universidade Federal do Rio de Janeiro, BRAZIL

## Abstract

The Formosan subterranean termite, *Coptotermes formosanus* Shiraki, is a costly invasive urban pest in warm and humid regions around the world. Feeding workers of the Formosan subterranean termite genetically engineered yeast strains that express synthetic protozoacidal lytic peptides has been shown to kill the cellulose digesting termite gut protozoa, which results in death of the termite colony. In this study, we tested if Melittin, a natural lytic peptide from bee venom, could be delivered into the termite gut via genetically engineered yeast and if the expressed Melittin killed termites via lysis of symbiotic protozoa in the gut of termite workers and/or destruction of the gut tissue itself. Melittin expressing yeast did kill protozoa in the termite gut within 56 days of exposure. The expressed Melittin weakened the gut but did not add a synergistic effect to the protozoacidal action by gut necrosis. While Melittin could be applied for termite control via killing the cellulose-digesting protozoa in the termite gut, it is unlikely to be useful as a standalone product to control insects that do not rely on symbiotic protozoa for survival.

## Introduction

The Formosan subterranean termite (FST), *Coptotermes formosanus* Shiraki, is a costly invasive urban pest in warm areas of high humidity, approximately 35° north and south of the equator, which includes the southeastern United States [1]. Workers of the FST have a symbiotic relationship with three species of protozoa living in their hind guts [2, 3]. Gut protozoa are vital for the survival of the termite colony since they facilitate digestion of lignocellulose, i.e., the major component in the diet of subterranean termites; loss of protozoa leads to death from starvation [4, 5, 6].

Previously, we provided proof of concept for a novel strategy for termite control using paratransgenesis [7, 8, 9, 10]. Paratransgenesis utilizes genetically engineered microorganisms to express and deliver gene products in a host organism [11]. Originally, paratransgenesis was used to eliminate pathogens from vector populations without killing the vectors [12]; however, we modified the approach to be applicable for termite control. Our latest product consists of genetically engineered yeast that expresses a synthetic protozoacidal lytic peptide (Hecate) coupled to a ligand that specifically binds the lytic peptide to protozoa [13]. When lytic peptide expressing yeast was fed to Formosan subterranean termites in a bait, workers lost all their gut protozoa within three weeks and the termite lab colonies died within two weeks thereafter [13]. Lytic peptides, a part of the innate immune system, are known to kill several microorganisms including protozoa [14], but are comparatively safe for higher eukaryotic cells [15]. The ligand increases target specificity and protects the gut tissue and prokaryotic symbionts from lytic peptide action [10, 13].

Since this product is highly specific to killing protozoa and its applicability is thus restricted to the control of subterranean termites, which rely on protozoa for survival, we decided to explore possibilities to develop a more broadly applicable paratransgenic system. Previous experiments showed that injection of low doses of Melittin into the hind gut of FST not only kills the protozoa but also leads to necrosis of gut tissue [9]. Melittin is a well-known cytolytic component of the honeybee venom [16] and is frequently applied in traditional Eastern and alternative medicine due to its antibacterial, antifungal and antitumor properties [17, 18]. Melittin is a strongly basic peptide and consists of 26 aminoacids with a molecular weight of 2.8 kDa [19], which is similar to other lytic peptides (Hecate and Cecropin B) previously shown to kill termite gut protozoa (Hecate and Cecropin, [9, 13]), Melittin is a pore forming peptide that induces membrane permeabilisation [20]. In this study, we tested if Melittin could be delivered into the termite gut via genetically engineered yeast and if the delivered Melittin killed termites via lysis of symbiotic protozoa in the gut of FST workers and/or destruction of the gut tissue itself.

## Materials and Methods

### Genetic engineering of yeast to express Melittin

The commercially available yeast *Kluyveromyces lactis* (New England Biolabs Inc. (NEB), MA, USA) was genetically engineered to express and secrete the lytic peptide Melittin (GIGAVLKVLTTGLPALISWIKRKRQQ-NH_2_, [21]). Codon optimized DNA sequences of Melittin for expression in *K*. *lactis* were obtained via Optimum Gene TM—Codon Optimization by GenScript Ltd., NJ, USA (5´GGC ATA GGC GCT GTG TTG AAA GTC CTT ACC ACA GGT TTG CCA GCA CTA ATA TCA TGG ATC AAG AGG AAA AGG CAG CAA3´). The Melittin gene was PCR amplified (primers: 5´GTAAAACGACGGCCAGT3´and 5´CAGGAAACAGCTATGAC3´) and the amplified fragment was cloned into the pKLAC2 expression vector (Cat. # E1000S, NEB) according to the manufacturer’s manual (https://www.neb.com/products/e1000-k-lactis-protein-expression-kit, for details see [13]). The pKLAC-Melittin constructs were cloned into competent *E*. *coli* cells (Cat# C2992, NEB). The constructs were isolated and linearized with *SacII* and the expression cassettes were introduced into the chromosome of *K*. *lactis* cells at the LAC4 locus according to the manufacturer’s protocols (https://www.neb.com/products/e1000-k-lactis-protein-expression-kit). Yeast strains were grown on yeast carbon base (YCB) agar medium containing 5mM acetamide at 30°C for 48 hrs. Single colonies were resuspended in 2 ml YPGal medium and incubated for 48 hrs at 30°C while shaking at 250 rpm. Yeast cells were harvested by centrifugation at 7000 X g for 30 sec and the culture supernatants were transferred to fresh tubes.

Correct integration of the expression cassettes into the genome of *K*. *lactis* cells was verified via PCR. Single colonies were suspended in 25μl of 1M sorbitol containing 2 mg/ml lyticase and incubated at 30°C. After 1 h, the lyticase-treated cells were lysed at 98°C for 10 min in a PTC-200 thermocyler (MJ Research Inc., Littletown, MA). PCR was performed according to the *K*. *lactis* instruction manual with the primers supplied with the *K*. *lactis* kit. Integration of the expression cassette at the LAC4 locus in the *K*. *lactis* genome was verified by the amplification of a 2.4 kb product (https://www.neb.com/products/e1000-k-lactis-protein-expression-kit).

Expressed Melittin was tentatively confirmed in the supernatant of engineered yeast cultures via NuPAGE^®^ Bis_Tris Precast Gels (Thermo Fisher Scientific Inc., Waltham MA) by the presence of a protein band in the 2.8 kDA region that did not appear in the controls. A non-lethal *K*. *lactis* strain expressing a far red fluorescent protein, mPlum (Clontech Laboratories Inc., CA, USA) was previously engineered by Sethi et al. [13] and used as control.

Toxic activity of the culture supernatants of yeast strains was measured against free-living aerobic protozoa of the species *Tetrahymena pyriformis* (Carolina Biological Supply Company, Burlington, NC). Fifty microliter of the culture supernatants was incubated with 50 μl of *T*. *pyriformis* culture with a concentration of 10 cells/μl. After 24 h, live protozoa were counted using a Sedgewick-Rafter cell (Pyser-SGI Limited, Kent, UK) under a microscope (Model: DMLB, Leica Microsystems Inc.) at 200 X magnification. The Melittin expressing yeast strain that was most effective against *Tetrahymena in vitro* was chosen for further evaluation in termite feeding assays.

### Termite feeding bioassays using genetically engineered yeast strains

Yeast strains were freeze-dried overnight using a lyophilizer and mixed separately at the rate of 1 part of yeast (0.25 mg) to 10 parts (2.5g) of α-cellulose powder and moistened with 6 ml of autoclaved tap water. Freeze drying does not impact viability and gene expression of yeast and yeast as a bait ingredient does not impact consumption [13].

Bait disks (1.5 x 0.5 cm) were cut out the mixture with a cork borer [13]. Triplicates of each feeding experiment were set up in Petri dishes (100 X 15 mm) with each dish containing one bait disk and 100 workers and 7 soldiers from a termite colony collected from New Orleans, LA. Three different treatments were applied, i.e., discs containing (1) Melittin-expressing yeast, (2) yeast expressing non-toxic red-fluorescent protein (mPlum) and (3) plain α-cellulose without any yeast. The Petri dishes were placed in a tray with moist paper towels and kept in an incubator at 27±2°C and 85% R.H. Each bait disk was hydrated with 300 μl autoclaved tap water every 48 h. The same experimental setup was repeated with a second termite colony from New Orleans, LA.

**Fig 1:**
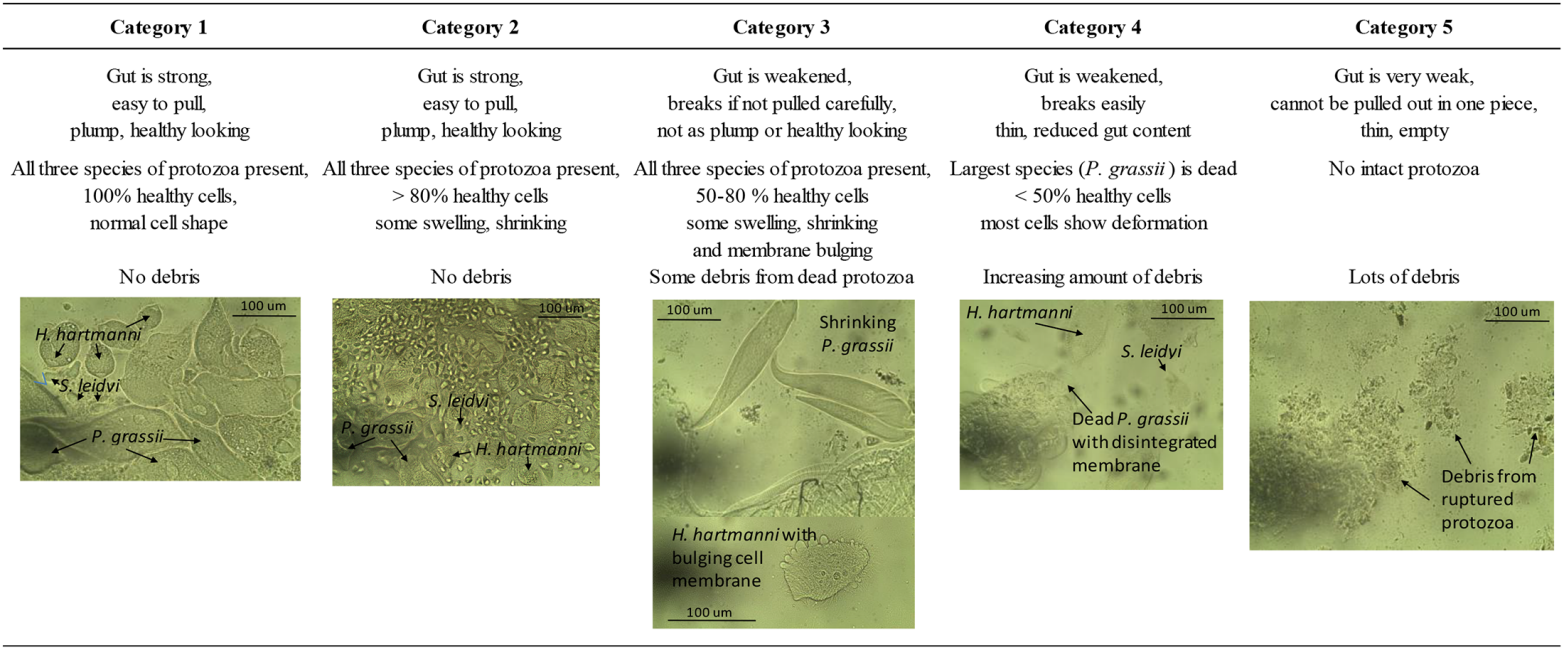
Description of the observed categories of gut and protozoa health of Formosan subterranean termite workers.

Five worker guts per treatment and replicate were extirpated using sterile forceps at days 2, 4, 6, 10, 13, 17, 20, 27 and 34. Hind guts were opened in an anaerobic glove box in sparged Trager U media on a glass slide using fine probes [10] and the integrity of each gut and status of protozoa was assessed on a scale from 1 (gut and protozoa are healthy) to 5 (gut lost integrity and protozoa, Fig 1) using both stereo (Model: MZ16, Leica Microsystems) and compound (Model: DMLB, Leica Microsystems) microscopes at 50–200 X magnification.

Uptake and retainment of *K*. *lactis* were qualitatively confirmed by the presence of blue colonies after plating the gut contents on *Kluyveromyces* differential medium [22]. Gene expression in the gut was confirmed as described in Sethi et al. [13].

### Feeding bioassays to assess gut integrity

Bait disks and feeding assays were prepared for one termite colony as described above to test effects of baits containing Melittin-expressing yeast versus a control of plain α-cellulose on gut integrity. Water for moistening the bait disks was laced with blue food color (Great Value^™^, Walmart, Baton Rouge, LA) to make gut contents visible. At days 1, 2, 7 and 20, five termite workers per treatment were observed under a stereo microscope (Model: MZ16, Leica Microsystems) for traces of colored gut content outside of the gut. Guts were also extirpated to test for traces of blue gut content in the body cavity. Results were recorded via a digital camera (Optronics, Goleta, CA) attached to the stereoscope.

### Statistics

Statistical analyses were performed with IBM SPSS Statistics Version 23 [23]. *P*-values < 0.05 were considered significant.

## Results and Discussion

Replicates had no effect on gut health (F = 1.19, P > 0.20, one-way ANOVA) and were thus combined. General Linear Model Analysis showed that colony origin, treatment and days of feeding had significant effects on gut and protozoa health (P ≤ 0.001) with no significant interactions (P ≥ 0.05) between these variables except for treatment*days (P ≤ 0.001, Table 1).

**Table 1:** General Linear Model (GLM) analysis of impact of colony origin, treatment (bait containing yeast expressing Melittin, yeast expressing mPlum or cellulose with no yeast) and days of feeding on the bait and their interactions (*).

GLM	Degrees of freedom	F-value	Significance
Colony	1	10.8	0.001
Treatment	2	193.77	<0.001
Days	12	58.85	<0.001
Colony * Treatment	2	2.18	0.12
Colony * Days	10	1.89	0.05
Treatment * Days	24	7.1	<0.001
Colony * Treatment * Days	20	0.98	0.49

**Fig 2:**
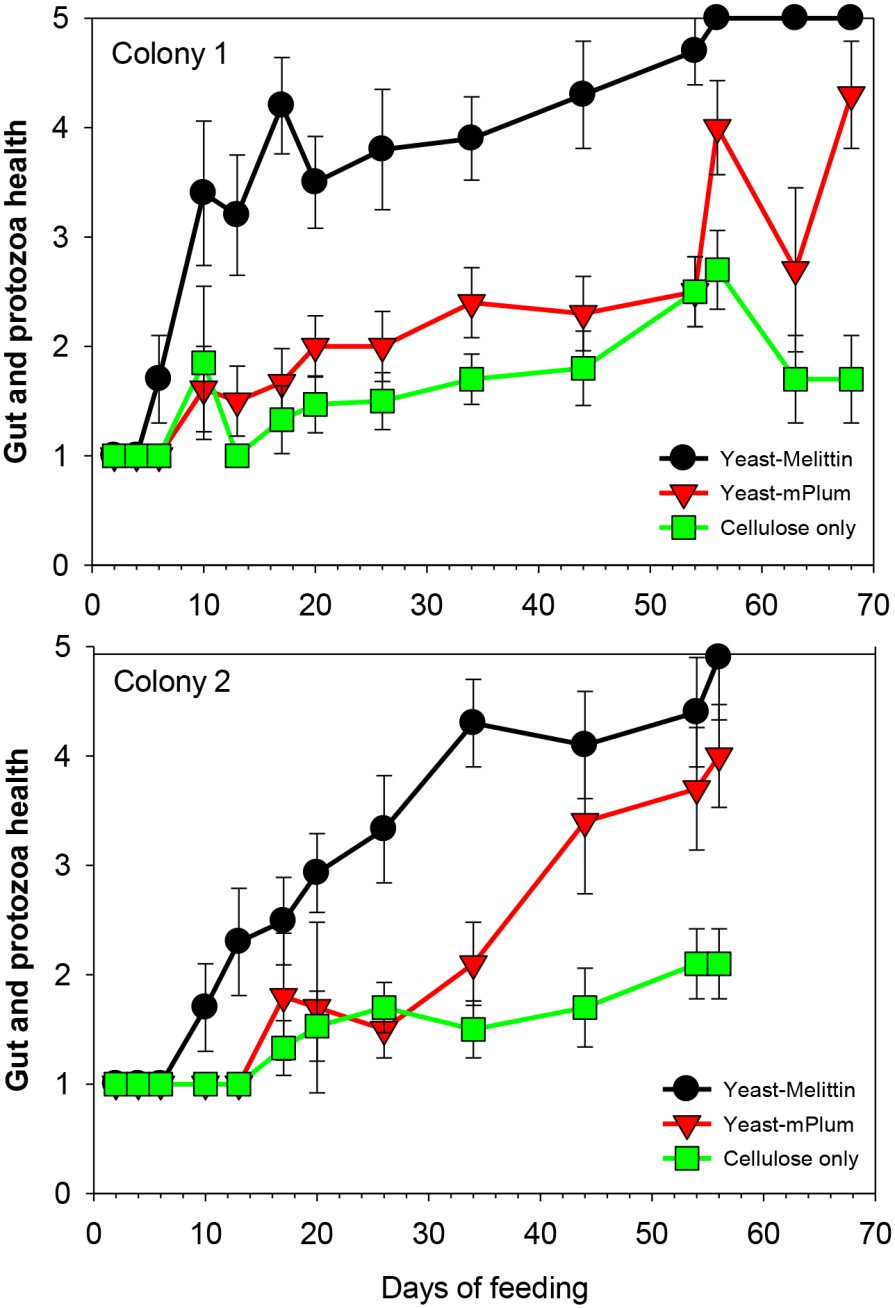
Changes in health of gut and protozoa of Formosan subterranean termite workers after feeding bait containing live yeast expressing Melittin, fluorescent protein (mPlum) or plain cellulose without yeast. Error bars represent 95% confidence intervals; non-overlapping confidence intervals signify significant difference among treatments.

All three treatments differed significantly in their effects on gut and protozoa health (P ≤ 0.001, Tukey HSD). As shown by the non-overlapping 95% confidence intervals in Fig 2, consumption of diet containing Melittin expressing yeast significantly decreased gut and protozoa health as early as day 6 (Colony 1) or day 10 (Colony 2) of feeding compared to both controls (cellulose bait with yeast expressing non-toxic fluorescent protein and plain cellulose). This delay might be a combination of the time it takes termites to ingest the critical amount of yeast, yeast slowly reviving from lyophylization in the termite gut, and the time until gene expression produces the necessary threshold of Melittin to show effects on protozoa. *In vitro* experiments have shown that it takes yeast revived from lyophilization 2–3 days to ramp up gene expression (pers. observation). Previous studies have shown that lytic peptide concentrations as low as 1 μM are sufficient to kill termite gut protozoa when injected into the hind gut of workers [10, 13]); however, the rate of ingestion of yeast cells, the percentage of cells producing Melittin, their rate of gene expression and, thus, the time needed to reach toxic levels of Melittin in the gut are not known.

Until day 36 (Colony 1) and day 44 (Colony 2), the controls do not differ from each other in their effect on gut health. However, towards the end of the experiment, the bait with yeast expressing mPlum caused decline in gut and protozoa health, but never caused complete defaunation in contrast to the loss of all protozoa caused by the treatment with Melittin-expressing yeast at day 54 and 56 for colony 2 and colony 1, respectively (Fig 2).

Although previous studies [13] have not indicated a detrimental effect of yeast unless it was expressing a lytic peptide, the longer duration of this experiment and twice higher concentration of yeast used in baits in this study may have exacerbated negative effects of the yeast intake and/or mPlum expression. Previous experiments have shown that the number of yeast cells increased 5-fold in the termite gut between week 2 and 3 of feeding [13]. It is conceivable that eventually the yeast overcrowds and outcompetes the natural gut flora leading to a decrease in gut health even without expressing a toxin. More fluorescent protein expressed by higher amounts of yeast in the bait than used in previous experiments also might alter the balance in the gut. This additional challenge to gut health even by yeast cells that do not express lytic peptides might benefit the overall goal of achieving termite control. However, the negative effect of Melittin expressing yeast on gut health was always significantly greater starting after the first week (expression ramps up) than the effect of yeast expressing fluorescent protein (Fig 2) until at least day 44 (colony 2) or day 56 (colony 1).

The results revealed that Melittin expressing yeast defaunates guts of termite workers, similar to what has been previously shown with other lytic peptides applied in their pure form [9, 10] or expressed by yeast [13]. However, the effect of Melittin was slower and less powerful than that of the synthetic lytic peptide Hecate and Hecate conjugated to a ligand that binds it to protozoa. While yeast expressing ligand-Hecate defaunated termites within three weeks [13], Melittin-expressing yeast took twice as long (56 days) to clear all protozoa from the gut despite its increased amount in the bait compared to the previous study when all workers feeding on ligand-Hecate bait died within five weeks, i.e., two weeks after complete defaunation [13]. In contrast, some termites fed on Melittin-expressing yeast were still alive on day 68 (colony 1) and day 58 (colony 2). Originally, we expected Melittin to be faster acting than ligand-Hecate due to the potential necrotic effect on the gut tissue observed previously [9]. However, the gut integrity was not compromised to a large degree as the dye feeding experiment showed.

**Fig 3:**
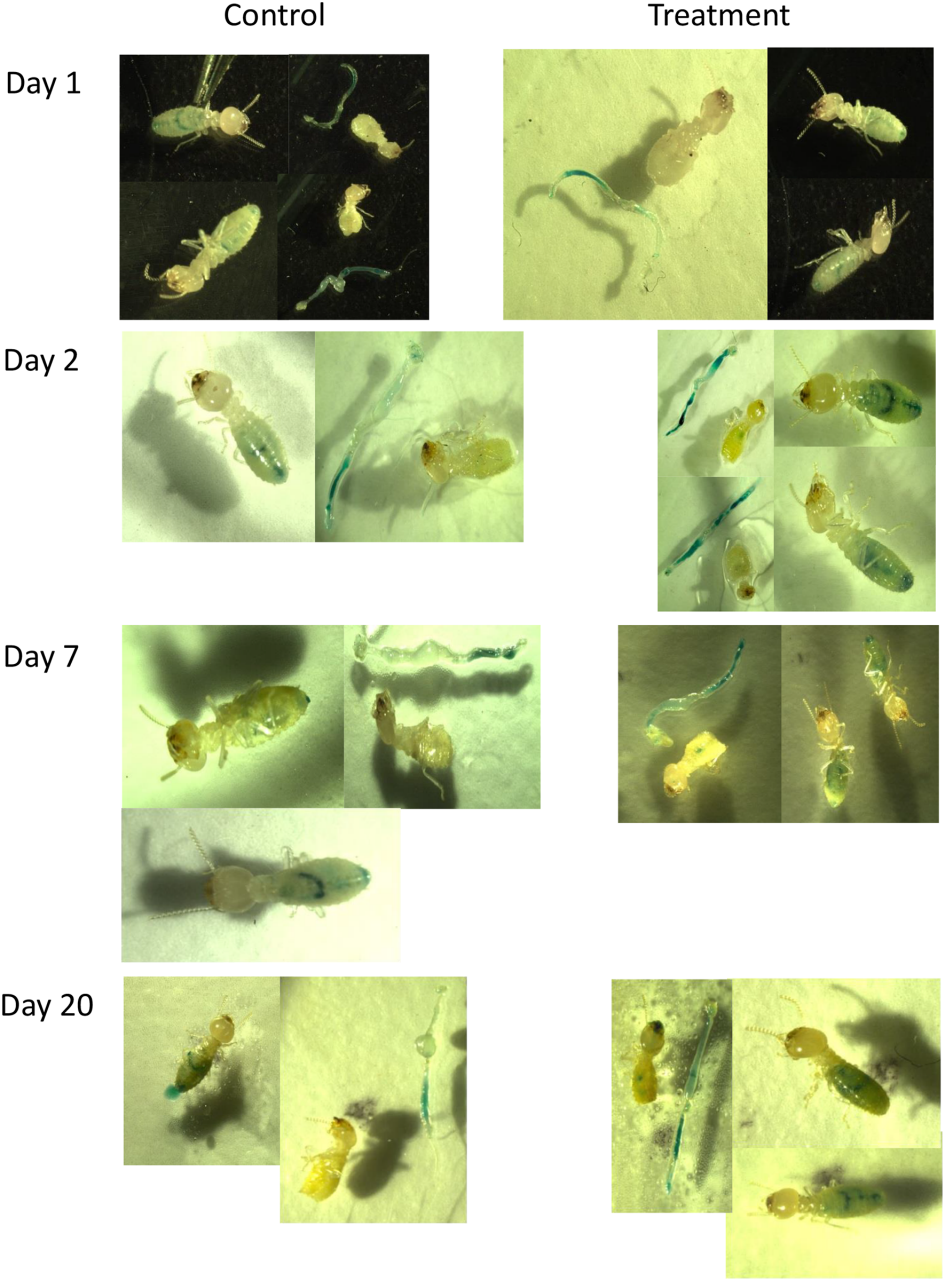
Test for gut integrity using workers fed with blue food dye on bait containing cellulose only (Control) or yeast expressing Melittin (Treatment).

Following ingestion of bait containing blue food dye, the blue dye was visible in the gut of termite workers in less than one day after feeding which confirmed the rapid uptake of bait (Fig 3). Without dissection there was no visible difference between dye distribution in workers fed on cellulose bait (control) or bait containing yeast expressing Melittin. However, extirpated guts of controls showed the most intensive color in the fore-and midgut sections, while the guts of termites ingesting yeast engineered to express Melittin had blue dye with almost equal intensity in the hind gut from day 2 after the start of the feeding assay. After removal of the gut, termites fed with Melittin expressing yeast also showed residual dye in their abdomen. This could have been due to either dye leakage *in vivo* or the guts rupturing during the gut pulling procedure because Melittin weakened the gut walls. In either case, differences between treatments and controls in dye distribution and presence of dye residue in the abdomen due to gut rupture suggest mechanical or metabolic changes associated with the ingestion of Melittin producing yeast. This supports the finding of the previous experiment based on observation of gut integrity and protozoa health (Fig 2), which showed that bait with yeast expressing Melittin caused noticeable negative effects within the first 6 days of feeding.

Previous experiments using injection of commercially available Melittin directly into the hind gut [9] resulted in necrosis of gut tissue visible as black tips of the abdomen attributed to cell/tissue death induced by Melittin. Necrosis was not observed in this study, possibly due to lower concentrations expressed by yeast cells in the gut compared to the injection of 50 μM Melittin and/or the absence of lysis enhancing other components of bee venom, such as phospholipase A2, an active ingredient in commercial Melittin [24].

In summary, Melittin expressing yeast did kill protozoa in the termite gut although the action took twice as long when compared to yeast expressing ligand-Hecate [13]. Under the conditions of this study, the expressed Melittin did not add a synergistic effect to the protozoacidal action via gut necrosis and thus, is unlikely to be useful as a standalone product to control insects that do not rely on protozoa. However, the weakening of the gut tissue could facilitate control when used in conjunction with sepsis inducing entomopathogens (such as *Serratia marcescens*, [25]).
